# Plant Growth-Promoting Endophytic Bacteria Isolated from *Miscanthus giganteus* and Their Antifungal Activity

**DOI:** 10.3390/microorganisms11112710

**Published:** 2023-11-05

**Authors:** Petra Lovecká, Gabriela Kroneislová, Zuzana Novotná, Jana Röderová, Kateřina Demnerová

**Affiliations:** 1 Department of Biochemistry and Microbiology, Faculty of Food and Biochemical Technology, University of Chemistry and Technology Prague, Technická 3, 166 28 Prague, Czech Republic; gabikrone@gmail.com (G.K.); zuzana.novotna@vscht.cz (Z.N.); demnerok@vscht.cz (K.D.); 2 Institute of Microbiology of the CAS, Vídeňská 1083, 142 20 Prague, Czech Republic; jana.roderova@biomed.cas.cz

**Keywords:** ACC deaminase, phosphorus solubilization, phytohormone, siderophore

## Abstract

Modern technologies can satisfy human needs only with the use of large quantities of fertilizers and pesticides that are harmful to the environment. For this reason, it is possible to develop new technologies for sustainable agriculture. The process could be carried out by using endophytic microorganisms with a (possible) positive effect on plant vitality. Bacterial endophytes have been reported as plant growth promoters in several kinds of plants under normal and stressful conditions. In this study, isolates of bacterial endophytes from the roots and leaves of *Miscanthus giganteus* plants were tested for the presence of plant growth-promoting properties and their ability to inhibit pathogens of fungal origin. Selected bacterial isolates were able to solubilize inorganic phosphorus, fix nitrogen, and produce phytohormones, 1-aminocyclopropane-1-carboxylic acid (ACC) deaminase, and siderophore. Leaf bacterial isolate *Pantoea ananat* is 50 OL 2 had high production of siderophores (zone ≥ 5 mm), and limited phytohormone production, and was the only one to show ACC deaminase activity. The root bacterial isolate of *Pseudomonas libanensis* 5 OK 7A showed the best results in phytohormone production (N6-(Δ2-isopentenyl)adenine and indole-3-acetic acid, 11.7 and 12.6 ng·mL^−1^, respectively). Four fungal cultures—*Fusarium sporotrichioides* DBM 4330, *Sclerotinia sclerotiorum* SS-1, *Botrytis cinerea* DS 90 and *Sphaerodes fimicola* DS 93—were used to test the antifungal activity of selected bacterial isolates. These fungal cultures represent pathogenic families, especially for crops. All selected root endophyte isolates inhibited the pathogenic growth of all tested fungi with inhibition percentages ranging from 30 to 60%. Antifungal activity was also tested in two forms of immobilization of selected bacterial isolates: one in agar and the other on dextrin-coated cellulose carriers. These results demonstrated that the endophytic *Pseudomonas* sp. could be used as biofertilizers for crops.

## 1. Introduction

Since the sixties of the twentieth century there has been talk of sustainable forms of agriculture, where in addition to the social and economic point of view the environmental aspect is emphasized. Methods and procedures have been sought and applied to preserve the environment and the agricultural landscape. One of the possible ways to promote plant growth and health may be the use of favorable interactions between plants and microorganisms that have been known for over a hundred years. New discoveries in the field of the plant microbiome stimulate the development of microbial inoculants, which can be used as biological fertilizers or plant protection agents against stress or disease. The bacteria-induced alterations of the plants offer many possibilities for biotechnological medicinal and agricultural applications. 

Ornamental grass (*Miscantus*) is a perennial ryegrass of photosynthetic type C4. It is unique among C4-type plants in that it maintains high photosynthetic activity at lower temperatures and is highly productive even in cold regions. In addition to their energy potential, growing ornamental plants also has a beneficial effect on soil because they increase the amount of organic matter and nutrients and support the microbial community [1]. In the conditions of Central Europe, diploid Chinese sedge (*Miscanthus sinensis* Andersson (1855)) or its triploid sterile hybrid with tetraploid sugar sedge (*Miscanthus sacchariflorus* (Maxim.) Franch.), giant sedge (*Miscanthus* × *giganteus* Greef & Deuter ex Hodkinson & Renvoiage), is most often cultivated. 

*Miscanthus* × *giganteus* is either moderately or highly tolerant of heat, drought, flooding, salinity (below 100 mM), and cool soil temperatures (down to −3.4 °C). Miscanthus grows relatively well in soils contaminated by metals, or by industrial activities in general. *Miscanthus* × *giganteus*’ perennial nature, its ability to grow on marginal land, its water efficiency, non-invasiveness, low fertilizer needs, significant carbon sequestration, and high yield have sparked significant interest among researchers, with some arguing that it has “ideal” energy crop properties. These properties can be reflected in an interesting microbial endophytic diversity.

Hudson et al. [2] define endophytic bacteria as bacteria that colonize the internal tissue of plants without causing infection or negative effects on the host. Endophytes colonize the interior of the plant body and can thus have a bigger impact on the host plant than rhizosphere bacteria. In addition, endophytes are ecologically adapted to the target niche and therefore can better overcome defensive reactions [3] than microorganisms originating from the rhizosphere. The advantage of using endophytic bacteria that return to the endophytic stage after product application is that they are better protected from biotic and abiotic threats coming from the plant’s surroundings. 

Many endophytic bacteria show plant-friendly properties in vitro, but only some of them maintain these properties in the plant, and only a small number of originally endophytic bacteria show a significant effect in promoting plant growth or are able to serve as a biocontrol agents in agricultural practice [4]. 

Endophytic bacteria, having both biodegradation and growth-promoting properties, are better at these effects than bacteria having only one of these properties. One of the protection mechanisms is the formation of ACC (1-aminocyclopropane-1-carboxylate) deaminase, which can directly reduce the level of ethylene due to stress conditions or promote plant growth by providing indolylacetic acid [5,6]. Endophyte bacteria increase the availability or supply of major nutrients that can also promote plant growth [7]. Nutrients, such as bound nitrogen, phosphorus, or iron, are usually deficient in agricultural soils.

The role of individual antimicrobial agents in the control of pathogens can vary with different bacterial endophytes. The degree of antimicrobial synthesis depends on nutritional factors (type of carbon source, trace elements, availability of other nutrients) as well as environmental factors. In a broader sense, we could include environmental protection and other stress conditions in biocontrol mechanisms [7,8].

Endophytes usually have antifungal activity but also have antibacterial effects, and sometimes they can suppress the proliferation of nematodes [9,10]. A microorganism used in biotechnological applications or for biocontrol in agriculture should be stable in changing conditions (pH, temperature, concentration of various ions) to efficiently colonize roots for the even distribution of, e.g., antimicrobial agents throughout the root system [11]. Endophytic microorganisms can inhibit the growth of fungal or bacterial pathogens by various mechanisms, including the production of antimicrobial substances, lytic enzymes, or siderophores. These mechanisms are similar to well-researched mechanisms that use rhizosphere bacteria. However, its study is more difficult due to the “hidden” way of life of endophytes in plant tissues [12,13,14]. So far, many endophytes synthesizing various antimicrobial substances have been described [15]. The most important producers are endophytic bacteria taxonomically classified in genera *Bacillus* and *Pseudomonas*. These endophytes are characterized by the ability to secrete antibiotic substances such as phenazine-1-carboxylic acid, phenazine-1-carboxamide, pyoluteorin, oomycin A, azomycin, and others [16]. Endophytic actinomycetes are also known which synthesize the antimicrobial agents munumbicin, kakadumycin, and coronamycin 1. For the production of antimicrobial substances that suppress the occurrence of diseases of agricultural crops, bacteria of the genus *Pseudomonas* stand out. Specifically, these are, for example, compounds produced by *Pseudomonas fluorescens* pyoluteorin and 2,4-diacytylphloroglucinol which inhibit the growth of tobacco root rot or other wheat diseases [16]. Next, the endophytic microorganism from *Phomopsis cassia* can be mentioned, which can synthesize an important antifungal metabolite of sesquiterpene structure, eliminating pathogenic micromycetes.

The main reason for the growing interest in endophytes is their possible use in biotechnological applications. In agriculture, endophytic microorganisms are mainly used to promote plant growth and biocontrol plant diseases [17]. Recently, bacterial endophytes have been used in different biotechnological sectors, such as bio-fertilizers to improve crop production and significantly reduce the chemical input into the environment, as well as in nanotechnology for the fabrication of various nanoparticles incorporated in different applications. Another possibility of using PGP bacteria is the immobilization of endophytic microorganisms in materials that can be used in organic agriculture to protect the soil from drying out, weeds, etc. [18,19].

In this study, we focused on determining the antifungal activity of several selected endophytic bacteria isolated from *Miscanthus giganteus*. These isolates were selected based on the results of Schmidt’s study [20]. The most common and most studied endophytes come from three strains (Actinobacteria, Proteobacteria, and Firmicutes), especially the genera *Azoarcus*, *Acetobacter* (*Gluconobacter*), *Bacillus*, *Burkholderia*, *Pantoea*, *Enterobacter*, *Herbaspirillum*, *Pseudomonas*, *Serratia*, *Stenotrophomonas*, and *Streptomyces* [4].

The innovativeness of the presented work lies in the extended and more detailed study of unique strains of endophytic microorganisms isolated from ornamental unconventional grass for antifungal protection and to support the survival and growth of this energy crop. Superior understanding of the native microbial endophytes in plants may clarify their capabilities and their potential in promoting plant growth and creating a sustainable system for crop production. 

## 2. Materials and Methods

### 2.1. Sampling and Isolation of Bacterial Endophytes

**Figure 1:**
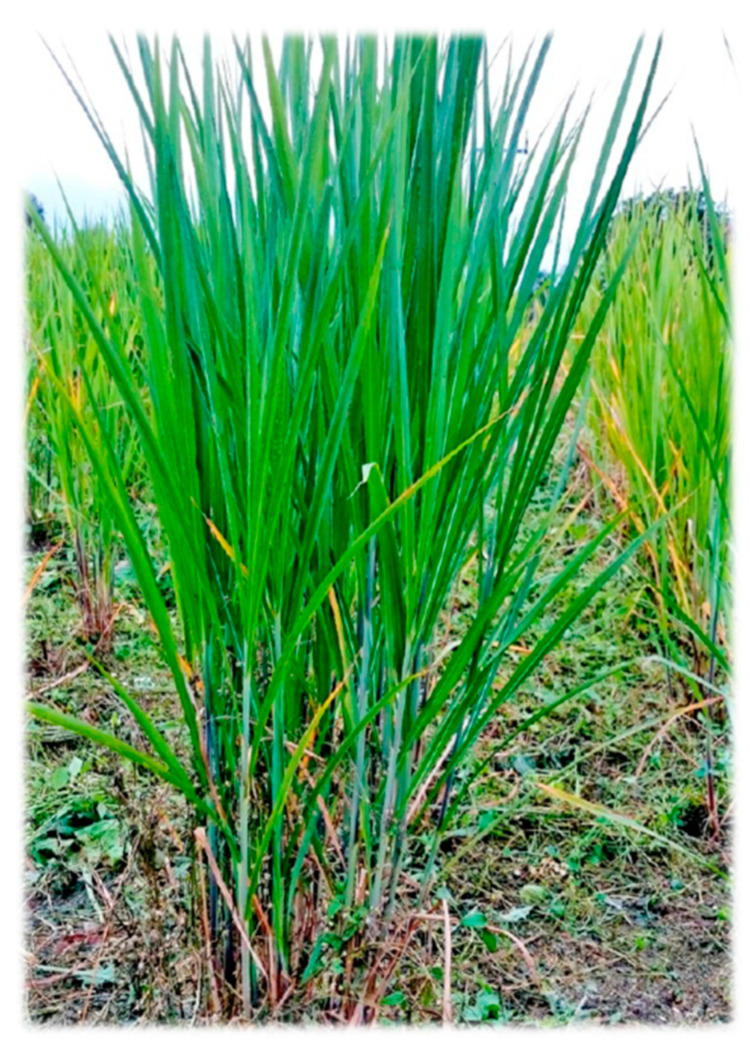
*Miscanthus giganteus*.

Shoots and roots of *Miscanthus gigantheus* were harvested in a field in Lukavec, Czech Republic (49°33′56″ N, 14°59′26″ E) in the summer, and five samples of leaves or roots from ten plants were preserved in sterile 0.01 M MgSO_4_ for a maximum of 2 h. The picture of the plant is shown in Figure 1.

Plant material was surface sterilized by a disinfecting agent for 7 min (5% sodium hypochlorite with the addition of 20 µL of 0.1% Tween 20), transferred to a 70% ethanol solution for 2 min, rinsed four times with sterile distilled water, and cut into small pieces (<0.5 cm). Endophytic bacteria were isolated using the method described by Schmidt et al. [18]. Approximately 2 g (fresh weight) of cut material was homogenized (Ultra-Turrax IKA-T10, max speed, 1 min) by thorough mixing under sterile conditions in 20 mL of sterile 12.5 mM potassium phosphate buffer (pH 7.1). This homogenate of *Miscanthus gigantheus* was used for inoculation of plates with diluted tryptic soy agar (5% TSA, 50% TSA) with cycloheximide (0.025 g L^−1^) and incubated for 48 h at 20 °C. Grown colonies were used for the isolation of bacteria.

Endophytes from roots were labeled by the designation “OK” in their code, respectively. Endophytes from leaves were labeled by the designation “OL” in their code, respectively.

### 2.2. Identification of Bacterial Isolates

Bacterial isolates were identified by matrix-assisted laser desorption/ionization time-of-flight mass spectrometry (MALDI-TOF MS) according to Koubek et al. [21] using an Autoflex Speed MALDI-TOF/TOF mass spectrometer and MALDI Biotyper 3.1 software (Bruker Daltonik, Bremen, Germany). MALDI-TOF MS scores between 2.300–3.000 mean a high probability of identification at the species level; 2.000–2.299 secure identification at genus level, with probable identification at species level; 1.700–1.999 mean probable identification at genus level. Selected bacterial isolates were identified via 16S rRNA gene analysis [20]. Genomic DNA was extracted from the purified isolates for identification. The 16S rRNA genes were amplified using the universal bacterial primers 27F (5′-AGAGTTTGATTCTGGCTCAG-3′) and 1492R (5′-GGTTACCTTGTTACGACTT-3′). The PCR was executed according to the following procedure: initial denaturation at 94 °C for 5 min; then 35 cycles at 94 °C for 1 min, 57 °C for 30 s, 72 °C for 30 s; and a final extension step at 72 °C for 7 min. The sequences assembled were analyzed by comparison with sequences in the GenBank database using the BLAST Alignment search tool (http://www.ncbi.nlm.nih.gov/blast/) (1 April 2018). The species of the isolate was determined as the species with the highest score in the BLAST results.

### 2.3. Detection of ACC-Deaminase Activity

The selected bacterial endophytes were tested for ACC deaminase activity with the colorimetric assay of 1-aminocyclopropane-1-carboxylate (ACC) based on ninhydrin reactions, according to Li et al. [22]. A decrease in absorbance at 570 nm compared to the control (non-inoculated nitrogen-defined medium with ACC) indicates that the given bacterial isolate exhibits ACC deaminase activity. The activity was rated as follows: low activity demonstrated OD_570nm_ ≤ 0.1, mean activity demonstrated OD_570nm_ 0.5–0.1, and high activity demonstrated OD_570nm_ ≥ 0.05.

### 2.4. Siderophore Detection

A siderophore production assay was performed by inoculating bacteria onto blue chrome azurol S (CAS) solid medium with hexadecyltrimethylammonium bromide (HDTMA). CAS/HDTMA complexly binds trivalent iron ions, resulting in a blue coloration. In this method, the detection of siderophores is based on the utilization of chromium azurol S in complex with iron [23]. Tested endophytic bacterial strains were inoculated into the CAS agar and incubated at 28 °C for 24 h. The development of a yellowish-orange halo around the colonies was taken as an indicator of siderophore production. Assessment was performed semi-quantitatively with three levels (low—zone ≤ 2 mm, medium—zone 2–5 mm, high—zone ≥ 5 mm). 

### 2.5. Phosphate Solubilization

Pikovskaya medium (agar), according to Jasim et al. [24], was used for the detection of solubilized calcium phosphate. The presence of a lysis circle around the bacterial colony was observed as an indicator of phosphate solubilization. Phosphate solubilization was detected by the clear zone. Similarly to siderophores, semi-quantitative evaluation (three levels: low—zone ≤ 2 mm, medium—zone 2–5 mm, high—zone ≥ 5 mm) of P-solubilization was used. 

### 2.6. Potential for Nitrogen Fixation

Colonies that appeared on the plates were selected and screened for nitrogenase activity on a yeast extract/mannitol/glutamate/succinate medium (g·L^−1^: yeast extract, 0.2; K_2_HPO_4_, 0.5; MgSO_4_.7H_2_O, 0.2; NaCl, 0.1; CaCO_3_, 0.5; mannitol, 10; sodium glutamate, 0.32; sodium succinate, 1.6; pH 7.2). The strain showed high nitrogenase activity when grown on this medium. The procedure for determining nitrogenase activity on agar and its interpretation is described by Dadarwal et al. [25]. Endophytic bacteria were inoculated into 5 mL of liquid LB medium and incubated at 28 °C with constant shaking for 24 h. An amount of 100 µL of the grown bacterial culture was inoculated into 5 mL of liquid medium for testing nitrogenase activity. Cultivation took place for 48 h at 28 °C with constant shaking. Subsequently, the optical density of individual samples was measured at a wavelength of 550 nm. The medium itself served as a negative control for testing nitrogenase activity. The activity was rated as follows: low activity demonstrated OD_517nm_ ≤ 0.05, medium activity demonstrated OD_517nm_ 0.5–0.1 and high activity demonstrated OD_517nm_ ≥ 0.1. 

### 2.7. Phytohormones Detection

Bacterial culture is prepared by cultivation for 48 h in LB medium, and suspension was filtered by a 0.2 µm bacterial filter. Indole-3-acetic acid (IAA) and the cytokinins N6-(Δ2-isopentenyl) adenine (iP) and N6-(Δ2-isopentenyl) adenosine (iPR) were quantified in cultural filtrates with ultra-high-performance liquid chromatography combined with mass spectrometry (UHPLC–MS/MS). This was carried out using an Acquity UPLC HSS T3 column (1.8 μm, 2.1 × 100 mm; Waters, Acquity, Milford, MA, USA) in a tandem mass spectrometer (AB Sciex, Qtrap 5500, Toronto, ON, Canada; electrospray ionization in positive mode), according to Hajšlová et al. [26]. Blank, sterile LB medium served as the control. 

### 2.8. Antifungal Activity of Endophytic Bacteria 

Dual culture in vitro assays were performed for all isolated bacterial strains [27]. It was carried out on Waksman’s agar adapted for mold growth (D–glucose 7 g·L^−1^, D–fructose 7 g·L^−1^, bacteriological agar 15 g·L^−1^, bacteriological peptone 5 g·L^−1^, meat extract 5 g·L^−1^). After sterilization, KH_2_PO_4_ 3.92 μM, NaCl 4.02 μM, KCl 1.8 μM, CaCl_2_ 100 μM, MgCl_2_ 134 μM, H_3_BO_3_ 5 μM, L-ascorbic acid 850 μM, Al_2_(SO_4_)_3_ 0.95 μM, K_2_SO_4_ 0.95 μM, and CuSO_4_ 0.25 μM were added. Antifungal activities of isolated endophytic bacteria were tested against four fungal species (*Botrytis cinerea* DS 90, *Fusarium sporotrichioides* DBM 4330, *Sclerotinia sclerotiorum* SS-1, *Sphaerodes fimicola* DS 93, from the microorganisms collection of Department of Biochemistry and Microbiology, UCT Prague). To evaluate the antagonist effect of different live bacteria, mycelia plugs from the edges of actively growing fungal cultures were placed in the center of a Petri dish containing agar. Four bacteria isolates were streaked on the same plates at an equal distance from the fungal inoculum. Plates with the fungal plug without bacteria were used as controls. Plates were incubated at 25 °C for 72 h to evaluate the inhibition activity of bacteria on the fungus. Zones of inhibition were calculated using the formula % of growth inhibition = ((C − T)/C)*100, where C is radial growth of the pathogen in the control plates (mm), and T is the radial growth of the pathogen in the test plates (mm). The experiment was repeated three times.

### 2.9. Immobilization of Endophytic Microorganisms in Agar and Testing of Their Antifungal Activities 

Endophytic bacteria were inoculated into 100 mL of liquid Waksman’s medium and incubated at 28 °C with constant shaking for 84 h. An amount of 0.5 mL of the grown bacterial culture was mixed with 2.5 mL of 1% technical agar. The mixture was poured over a Petri dish with PDA (Potato Dextrose Agar). On this Petri dish, two wells with a diameter of 7.5 mm were excavated and filled with agar with fungal cultures of two of the four tested microscopic fungi (*Botrytis cinerea*, *Fusarium sporotrichoides*, *Sclerotinia sclerotiorum*, *Sphaerodes fimicola*). A Petri dish poured over with a mixture of 0.5 mL of sterile water and 2.5 mL of technical agar and applied fungal cultures served as a control. The Petri dishes were incubated at a temperature of 26 °C, the evaluation took place after 72 h. Antifungal activity was manifested by a smaller growth of the fungus compared to the control.

### 2.10. Immobilization of Endophytic Microorganisms into Dextrin-Coated Cellulose Carriers and Testing of Their Antifungal Activities

Endophytic bacteria were inoculated into 100 mL of liquid Waksman’s medium and incubated at 28 °C with constant shaking for 84 h. An amount of 0.5 mL of the grown bacterial culture was applied to sterile filter paper, which was dried at 50 °C. Subsequently, 0.6 mL of 10% dextrin was applied to this paper. The paper was dried again at a temperature of 50 °C. The paper with immobilized endophytic microorganisms was placed on a Petri dish with PDA, on which two wells were excavated and filled with agar with fungal cultures of two of the four tested microscopic fungi (*Botrytis cinerea*, *Fusarium sporotrichoides*, *Sclerotinia sclerotiorum*, *Sphaerodes fimicola*). A Petri dish with the applied fungal cultures was covered with filter paper, to which sterile water was applied as a control. The Petri dishes were incubated at a temperature of 26 °C and the evaluation took place after 72 h. Antifungal activity was manifested by a smaller growth of the fungus compared to the control.

## 3. Results 

### 3.1. Isolation and Identification of Endophytic Bacteria

This work follows on from the research results of Schmidt et al. [20], which show the positive importance of bacterial endophytic isolates for plant growth. In our study, we focused on a more detailed description of five selected isolates, namely on testing their antimicrobial activity in free and immobilized form. Several selected bacterial endophytes were isolated from the *Miscanthus giganteus* plant, one bacterial isolate from the leaves (OL labels) and four bacterial isolates from the roots (OK markings). All bacterial strains were identified by MALDI-TOF MS. With MALDI-TOF MS scores greater than 2.000, a majority of isolates could securely be identified at the genus level. Gammaproteobacteria were by far most frequently isolated, with *Pantoea ananatis* (Enterobacteriales) present in leaves, and *Pseudomonas libanensis* in roots, and other members of the genus *Pseudomonas* residing in roots. With MALDI-TOF MS scores between 2.300–3.000, there is a high probability of identification at the species level; 2.000–2.299 secures identification to the genus level, and probable identification to the species level; 1.700–1.999 probable identification at the genus level. Selected isolates with the best PGP properties were subjected to 16S rRNA analysis, and the isolates were identified as *Pseudomonas libanensis* and *marginalis*, *Pantoea ananatis*, and *Variovorax paradoxus* [20]. 

### 3.2. Plant Growth-Promoting Potential of Bacterial Isolates

The results presented by Schmidt et al. [20] show that endophytic bacteria can increase plant biomass and additionally produce bioactive compounds that have the potential to be used as sustainable alternatives to traditional fertilizers and pesticides. The plant growth-promoting potential of five selected isolates from plant tissue was characterized in vitro by determining qualitatively and/or quantitatively ACC deaminase activity, phosphate solubilization ability, iron carrier siderophore production, nitrogen fixation (Table 1), and production of phytohormones (Table 2). The results shown are a selection of large published studies by Schmidt et al. [20]. Four root and one leaf isolates with different PGP characteristics were selected for the following experiment, testing antifungal activity. 

The ability to fix nitrogen was monitored for all isolates, with the root isolates to a greater extent than the leaf isolate. Isolates 50 OK 3 and 5 OK 7A were identified as phosphate-solubilizing microbes, according to their ability to solubilize inorganic phosphorus. The leaf strain 50 OL 2 was found to be positive for the production of ACC deaminase; ACC deaminase activity was not determined for root isolates. The endophytic strains leaf 50 OL 2 and root 50 OK 6 were able to produce siderophores, as confirmed by a change of color. The root bacterial isolate 5 OK 7A showed the best results for phytohormone production and was able to produce all tested phytohormones (N6-(2-isopentenyl) adenine, N6-(2-isopentenyl) adenosine, and indole-3-acetic acid).

### 3.3. Antifungal Activity of Bacterial Isolates 

**Figure 2:**
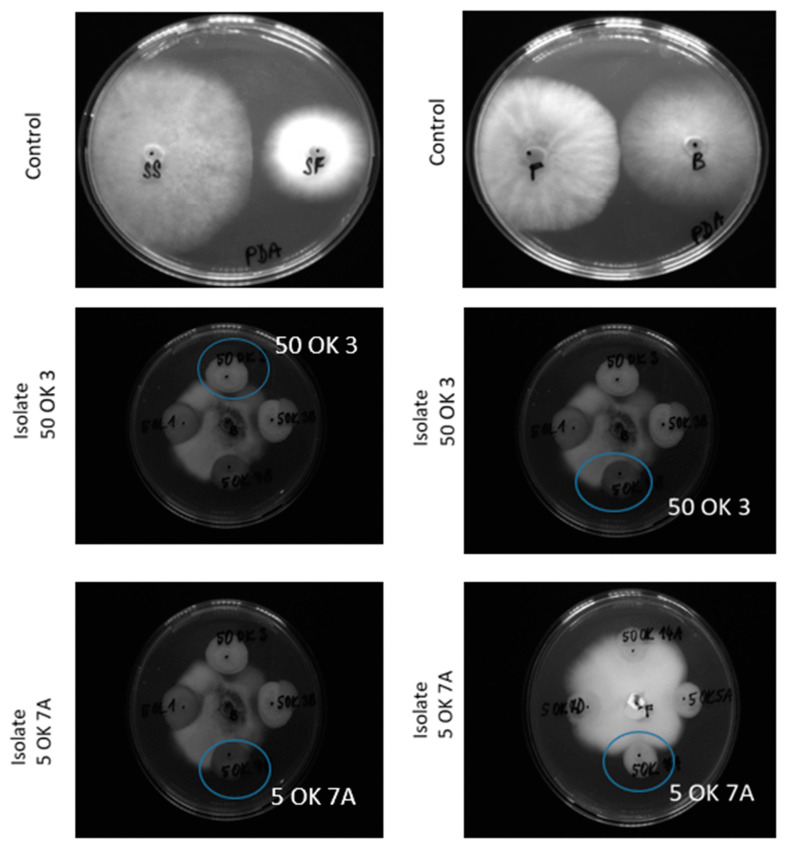
In vitro antifungal activities of the isolated endophytic bacterial strains (50 OK 3, 50 OK 6, 50 OK 7A, and several not described in this study). SS, *Sclerotinia sclerotiorum*; SF, *Sphaerodes fimicola*; *F*, *Fusarium sporotrichoides*; B, *Botrytis cinerea*. Blue circle detects positive result (scale bar 9 cm Petri dish).

**Figure 3:**
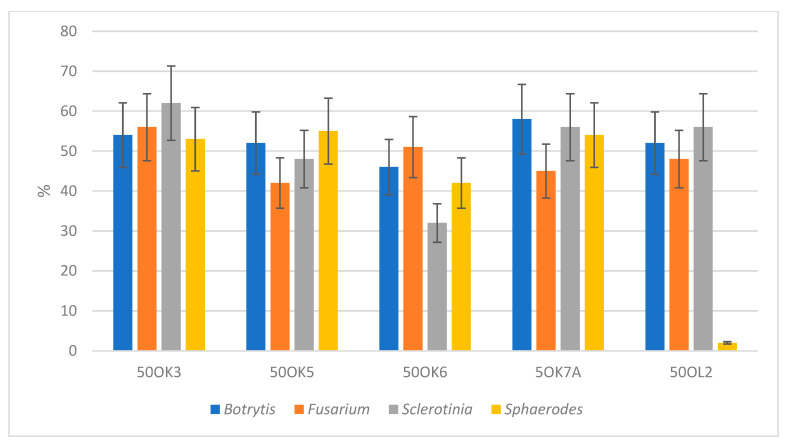
Antifungal activities measured as size of the zones of pathogenic fungi. Zones of inhibition are expressed as percentages.

The next potential of endophytic bacteria is their possible inhibition of fungal plant pathogens and reduction of the incidence of fungal diseases. In our case, we focused on the antifungal activity (measurement of fungal pathogen growth inhibition) of already isolated bacteria, with the possibility of their use in organic farming, especially in the protection of crops from Fusarium wilt. Four fungal cultures—*Fusarium sporotrichioides* DBM 4330, *Sclerotinia sclerotiorum* SS-1, *Botrytis cinerea* DS 90, and *Sphaerodes fimicola* DS 93—were used to test the antifungal activity (Figure 2). These cultures were selected because they are representatives of known pathogenic fungi families, especially for crops. *Fusarium sporotrichoides*, for example, causes disease in pastinaca (*Pastinaca sativa*). This mold is a producer of phytotoxins [28]. *Sclerotinia sclerotiorum* is a common non-specific soil pathogen affecting more than 400 species of plants, e.g., cabbage, strawberry, lettuce, bean, etc. The symptoms of the attack include plant tissue wetting, coatings, and black structures on the surface of plants. *Botrytis cinerea* is a necrotrophic fungus that affects many plant species; its most notable host is grapevine. The data represented in Figure 2 show the in vitro antifungal activities of isolated bacterial endophytes against four phytopathogens. In general, root isolates exhibit more pronounced antifungal activity than leaf isolates (Figure 3). Four of the root endophytes (50 OK 3, 50 OK 6, 5 OK 7A—*Pseudomonas libanensis*, *Ps. marginalis*, 50 OK 5—*Variovorax paradoxus*) were able to inhibit growth of all four tested fungi (*Botrytis cinerea*, *Fusarium sporotrichioides*, *Sclerotinia sclerotiniorum*, *Sphaerodes fimicola*). Isolate 50 OL 2 inhibited only *Botrytis cinerea*, *Fusarium sporotrichioides*, *Sclerotinia sclerotiniorum*. Antifungal activity is usually not species-specific. Endophytic bacteria in most cases inhibited the growth of *Botrytis cinerea* and were also very effective acting against *Fusarium sporotrichoides*. On the other hand, the growth of the fungus *Sphaerodes fimicola* was not inhibited by any leaf isolate. The highest inhibition percentages were achieved due to treatment with bacterial root isolate 50 OK 3 (*Pseudomonas libanensis*). 

There are many works describing the antifungal activity of selected species [29,30] with the exception of *Variovorax paradoxus*, which is not much known from this perspective. The three samples which had the highest antifungal activity under all conditions tested were all bacteria belonging to the group *Pseudomonas*.

### 3.4. Evaluation of Antifungal Activity of Immobilized Endophytic Isolates 

**Figure 4:**
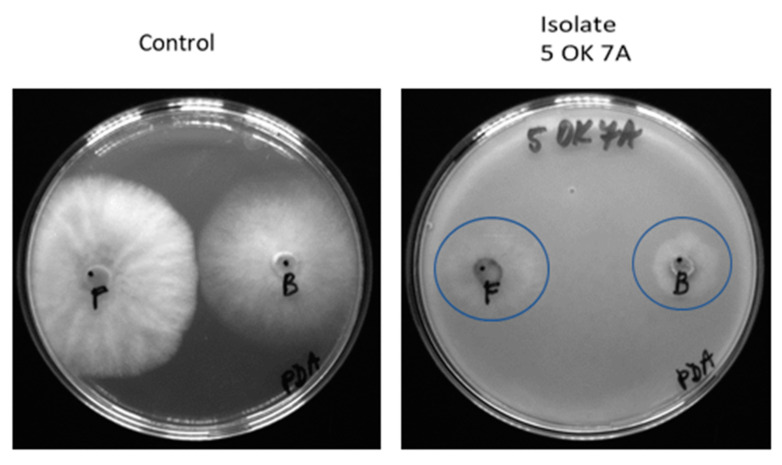
Antifungal activity of an endophytic microorganism 5 OK 7A immobilized in agar on the fungus *Fusarium sporotrichoides* (F) and *Botrytis cinerea* (B) on PDA; the area of fungal growth is marked in blue (scale bar 9 cm Petri dish).

**Figure 5:**
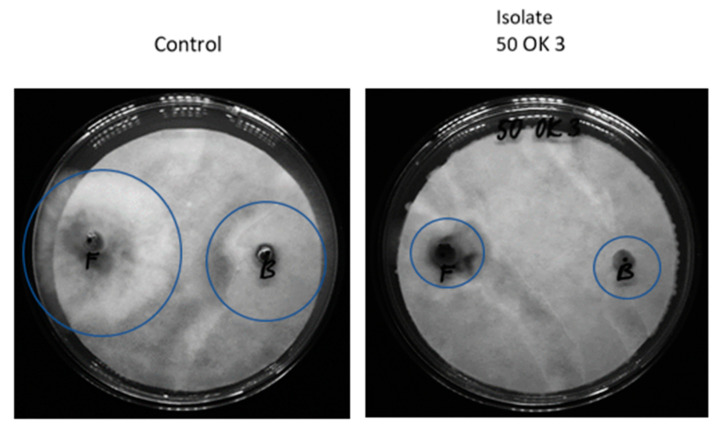
Antifungal activity of immobilized endophytic microorganism 50 OK 3 on *Fusarium sporotrichoides* (F) and *Botrytis cinerea* (B) in dextrin-coated cellulose carriers; the area of fungal growth is marked in blue (scale bar 9 cm Petri dish).

In another experiment, the antifungal activity of strains immobilized in different carriers was confirmed. Agar was chosen as a carrier for immobilized samples due to the simplicity of testing and easy evaluation for the first screening test. Cellulose was chosen due to its easier application in field conditions. Endophytic microorganisms immobilized in agar had very good inhibitory activity on all fungi growing on PDA (Table 3, Figure 4) and dextrin-coated cellulose carriers (Table 4, Figure 5). The only exceptions were root isolates 50 OK 5 (*Variovorax paradoxus*) and 50 OK 6 (*Pseudomonas marginalis*), which did not inhibit the growth of one of the four fungi tested in dextrin-coated cellulose carriers. Antifungal activity in both cases of immobilization was also demonstrated in the leaf isolate 50 OL 2, which did not show activity in its non-immobilized form.

## 4. Discussion

The main task of this work was to screen the properties of naturally occurring strains of endophytic bacteria and their applicability in practice. Several interesting bacterial genera have been isolated. *Pantoea ananatis*, isolated from leaves, is a common species with a worldwide occurrence. It is airborne and can be found in soil, water, and on plant surfaces as an epiphyte. It is an endophyte for many plant species and may also be a pathogen [30]. Most of the root isolates studied in this study belong to the Pseudomonas group: *Pseudomonas*, *Ps. libanensis*, *Ps. marginalis*. The MALDI-TOF MS method is not able to accurately distinguish between individual species in this group, and therefore species identification is only indicative. Bacteria from this group can be found almost anywhere in the world, even in plant and animal bodies where they are part of the common microflora but can act as pathogens [31].

Production of ACC deaminase is one of important characteristic of plant growth-promoting endophytes, since ACC deaminase cleaves ACC, the immediate precursor of the plant hormone ethylene [12]. ACC deaminase activity was demonstrated for *Pantoea ananatis* bacteria. This finding agrees with the published results, where only a fraction of strains showed this activity [32,33,34]. For *Pseudomonas libanensis*, ACC deaminase activity was not demonstrated. This finding agrees with the results published in the literature, where it was shown that some strains may have but others may not have this type of activity [34]. Among the isolated bacteria of the *Pseudomonas* group originating from the roots, it was possible to prove ACC deaminase activity in only a single isolate. This is not in direct contradiction to the published results in the literature; although this activity has been demonstrated in the published results, it is so low that it is unlikely to be proven with the method used [32,33,34,35]. *Variovorax paradoxus* did not show ACC deaminase activity when tested, although this activity was described in the literature [36]. 

Regarding phosphate solubilization, published data show that some strains of the bacterium *Pantoea ananatis* can show the ability to solubilize phosphate [33,37]. This finding agrees with the results obtained with our isolates, where this ability occurs only occasionally. *Ps. libanensis* demonstrated this ability, but it always comes from the isolates from the rhizosphere [35]. Pseudomonads from the group *Ps. fluorescens* were able to solubilize phosphate in most cases, but there were large differences in the degree of solubilization among them [34].

In general, it can be said that isolates from roots are able to solubilize phosphate. This is quite logical, because when the bacterium is associated with the roots, it is a beneficial property that allows the plant to obtain phosphate, whereas above ground, where there is no insoluble phosphate, this property is meaningless.

The isolates of the bacteria *Pantoea ananatis*, *Pseudomonas libanensis*, and *Ps. marginalis* showed the ability to produce siderophores, which results agree with those in the literature [32,35]. *Variovorax paradoxus* shows no ability to produce siderophores, but this ability is confirmed in most published works [38,39]. Interestingly, this property was induced in *Variovorax* strains in the presence of metals. Siderophores produced by bacteria have a great affinity for iron, forming a complex that can be assimilated by plants [40]. Siderophores produced by *Variovorax* could promote a plant’s metal uptake, unless it is not clear if siderophores promote or reduce a plant’s metal uptake, and there are examples of both behaviors [41].

Nitrogen is an essential and vital element for normal growth and developments of plants. All samples were able to fix atmospheric nitrogen. The activity of the leaf isolate was the lowest. It has been shown in the literature that they may have this ability [37]. The nitrogen-fixing potential of each bacterial isolate was assessed by its ability to grow on nitrogen-free minimal medium. Although not directly tested for the presence of nitrogenase, Dadarwal et al. [25] proved in their work that all bacteria that were able to grow on the medium for testing nitrogenase activity showed this ability. 

The results achieved reveal that all isolated strains produce cytokinin in the form of N6-(2-isopentenyl)adenine (iP), which may not be surprising since N6-(2-isopentenyl)adenosine is a part of the tRNA of all bacteria and eukaryotes, and the formation of iP depends only on the presence of two common enzymes [42,43]. If we stick to the division of strains of the bacterium *Pantoea ananatis*, as suggested in their work by Kido et al. [43], which is based on the relationship between phytohormone production and pathogenicity, then three of the samples probably belong to the II. group that produces both auxin and cytokinins but causes no disease, and the remaining samples belong to the III. group that is characterized by the absence of genes for the production of indolyle-3-acetic acid (IAA), but can be pathogenic to some plant species. In *Pseudomonas savastanoi*, genes for IAA production are mostly present on plasmids, and therefore IAA production is highly variable in this species [44]. In root pseudomonads from the group *Pseudomonas*, the production of IAA is dependent on a yet be precisely identified pathway that is tryptophan-dependent [40]. The production of phytohormones is highly variable in different strains of the bacterium *Variovorax paradoxus* and strains with high productivity are known, as well as strains that do not have the pathway for auxin synthesis at all [45].

Endophytic microorganisms can inhibit the growth of fungal or bacterial pathogens by various mechanisms, including the production of antimicrobial substances, lytic enzymes, or siderophores. These mechanisms are similar to well-studied mechanisms used by rhizosphere bacteria. Some bacteria produce enzymes (chitinases, cellulases, β-1,3-glucanases, proteases, or lipases) that can lyse part of the cell walls of many pathogenic fungi. Biocontrol activity against a number of pathogenic fungi has been found for PGPBs (plant growth-promotion properties bacteria) that synthesize one or more of these enzymes, e.g., against the fungi *Botrytis cinerea*, *Sclerotium rolfsii*, *Fusarium oxysporum*, *Rhizoctonia solani*, *Pythium ultimum*, and the genus Phytophthora [13,46]. 

The plant *Miscanthus giganteus* is not often attacked by many pathogens of fungal origin, although more of them are beginning to appear with the spread of its monocultural cultivation [47]. It is possible that the plant itself forms no antifungal agents and relies on fungal pathogens for the protection of endophytic microorganisms with antifungal properties themselves. The immobilization of bacterial isolates into different material carriers mostly preserved the antifungal activities of the tested bacteria. When comparing all results, two bacterial isolates appear to be the most suitable for practical use, *Pantoea ananatis* 50 OL 2 and *Pseudomonas libanensis* 5 OK 7A, which showed both the most prominent growth-promoting properties and antifungal activity. Immobilized microbial biostimulants have the potential to be a long-term and successful method for reducing abiotic and also biotic stressors While recent advancements and laboratory studies have revealed the positive activities of plant-associated microorganisms, the efficacy of microbial biostimulants is yet to be successfully validated in field experiments [19].

## Figures and Tables

**Table 1:** Plant growth-promoting potentials of selected bacterial isolates from the leaves and roots of *Miscanthus giganteus*.

Isolates from Plant Tissue	Identification	ACCd	P-SOL	SID	NITRO
50 OK 3	*Pseudomonas libanensis*	-	+++	+	+++
50 OK 5	*Variovorax paradoxus*	-	-	-	++
50 OK 6	*Pseudomonas marginalis*	-	+	+++	++
5 OK 7A	*Pseudomonas libanensis*	-	+++	+	++
50 OL 2	*Pantoea ananatis*	+	++	+++	+

50, isolation of z 50% TSA; 5, isolation from 5% TSA; OK, isolates from the roots; OL, isolates from the leaves; ACCd, ACC-deaminase: ‘+’ low activity demonstrated (OD_570nm_ ≤ 0.1); ‘-’ no activity; P-SOL, phosphate solubilization: ‘+’ low activity demonstrated (zone ≤ 2 mm); ‘++’ mean activity demonstrated (zone 2–5 mm); ‘+++’ high activity demonstrated (zone ≥ 5 mm); ‘-’ no activity; SID, production of siderophores: ‘+’ low production demonstrated (zone ≤ 2 mm); ‘+++’ high production demonstrated (zone ≥ 5 mm); ‘-’ no production; NITRO, nitrogen fixation: ‘+’ low activity demonstrated (OD_517nm_ ≤ 0.05); ‘++’ mean activity demonstrated (OD_517nm_ 0.05–0.1); ‘+++’ high activity demonstrated (OD_517nm_ ≥ 0.1).

**Table 2:** Amount of phytohormones (ng·mL^−1^) produced by bacterial isolates from the leaves and roots of *Miscanthus giganteus*.

Isolates from Plant Tissue	Identification	Phytohormones (ng·mL^−1^)		
		iP	iPR	IAA
50 OK 3	*Pseudomonas libanensis*	5.5	n.d.	n.d.
50 OK 5	*Variovorax paradoxus*	3.8	n.d.	n.d.
50 OK 6	*Pseudomonas marginalis*	3.7	n.d.	n.d.
5 OK 7A	*Pseudomonas libanensis*	11.7	8.9	12.6
50 OL 2	*Pantoea ananatis*	1.1	n.d.	n.d.

Prefix 50, isolation from 50% TSA; 5, isolation from 5% TSA; OK, isolates from the roots; OL, isolates from the leaves; iP, *N*6-(2-isopentenyl)adenine; iPR, *N*6-(2-isopentenyl)adenosine; IAA, indole-3-acetic acid; n.d.—not detected.

**Table 3:** Evaluation of antifungal activity of endophytic isolates immobilized in PDA agar.

Isolates from Plant Tissue	Identification	*Botrytis cinerea*	*Fusarium sporotrichioides*	*Sclerotinia sclerotiniorum*	*Sphaerodes fimicola*
50 OK 3	*Pseudomonas libanensis*	+	+	+	+
50 OK 5	*Variovorax paradoxus*	+	+	+	+
50 OK 6	*Pseudomonas marginalis*	+	+	+	+
5 OK 7A	*Pseudomonas libanensis*	+	+	+	+
50 OL 2	*Pantoea ananatis*	+	+	+	+

50, isolation from 50% TSA; 5, isolation from 5% TSA; OK, isolates from roots, OL, isolates from leaves; ‘+’ activity proven;

**Table 4:** Evaluation of antifungal activity of endophytic isolates immobilized in dextrin-coated cellulose carriers.

Isolates from Plant Tissue	Identification	*Botrytis cinerea*	*Fusarium sporotrichioides*	*Sclerotinia sclerotiniorum*	*Sphaerodes fimicola*
50 OK 3	*Pseudomonas libanensis*	+	+	+	+
50 OK 5	*Variovorax paradoxus*	+	-	+	+
50 OK 6	*Pseudomonas marginalis*	+	+	-	+
5 OK 7A	*Pseudomonas libanensis*	+	+	+	+
50 OL 2	*Pantoea ananatis*	+	+	+	+

Prefix 50, isolation from 50% TSA; OK, 5, isolation from 5% TSA; OK, isolates from roots; OL, isolates from leaves; ‘+’ activity proven; ‘-’ activity not proven.

## Data Availability

All data are freely available within this article. Purified cultures of each of the bacterial strains were deposited at the microorganisms collection of Department of Biochemistry and Microbiology, UCT Prague) on January 2018.
